# Microcystic Adnexal Carcinoma. A Rare Entity

**DOI:** 10.4317/jced.61440

**Published:** 2024-04-01

**Authors:** Angela Sada-Urmeneta, Marta Benito-Anguita, Carolina Agra, Dafne Gascon-Alonso, Marc Agea-Martinez, Carlos Navarro-Cuellar, Juan-Jose Verdaguer

**Affiliations:** 1 Maxilofacial Surgery Department, Hospital Gregorio Marañon. Madrid, Spain; 2 Pathology department, Hospital Gregorio Marañon. Madrid, Spain

## Abstract

**Background:**

Microcystic adnexal carcinoma is a rare malignant tumour derived from sweat glands, locally aggressive, but with low rate of lymphatic or metastatic spread. Tends to affect the deep dermis, without affection of epidermis. Surgery remains as the first line treatment.

**Case Report:**

We present a case of a 46-year-old woman with a slow growing lesion of the upper lip, with biopsy diagnosis of microcystic adnexal carcinoma. She underwent a resection and reconstruction with local advancement flaps. The final anatomopathological study showed an adnexal epithelial neoplasm with imprecise borders, poorly delimited, non-encapsulated, growing in plaque-like formation from the superficial dermis into the adipose tissue, perineural invasion, without epidermal infiltration.

**Discussion:**

It is an extremely rare malignant tumour, appearing as a solitary papule or plaque affecting the central face, that often affect middle-aged caucasic, female patients. Usual local aggressive nature, characterized by small nests and strands of cells in deep dermis and perineural-invasion images, absent in superficial tissue. An incisional biopsy is need to make a correct diagnosis. Due to its rarity there is no consensus on the best management and follow-up. The microcystic adnexal carcinoma should be taken into consideration in the differential diagnosis.

** Key words:**Microcystic adnexal carcinoma, lip, histopathology.

## Introduction

Microcystic adnexal carcinoma is a rare entity, with only few cases published in literature since its first description by Goldstein *et al*. in 1982 (1,2). It is a malignant tumour derived from sweat glands (1,3,4) that usually appears in caucasian, middle-aged, female patients (5), as a slow-growing papula or plaque in the central face (1). It has been related to ultraviolet radiation, ionizing radiation and immunosuppression as risks factors; without any solid evidence (2,6). Although its local aggressiveness, lymphatic or metastatic spread is very rare (2,4,7).

The microcystic adnexal carcinoma tends to affect deep dermis and has high rate of perineural invasion (4), that is why an incisional biopsy is needed to make an accurate diagnosis.

Surgery remains as the first-choice treatment, either wide resection with margins or MOHS surgery, in order to avoid its high recurrence rate (4,7,8). If surgery cannot be done or free margins are not possible, radiotherapy has been proposed as adjuvant therapy or even monotherapy; with no consensus on its efficacy nor its protocol of treatment (4,7,9). The role of chemotherapy remains controversial.

## Case Report

**Figure 1:**
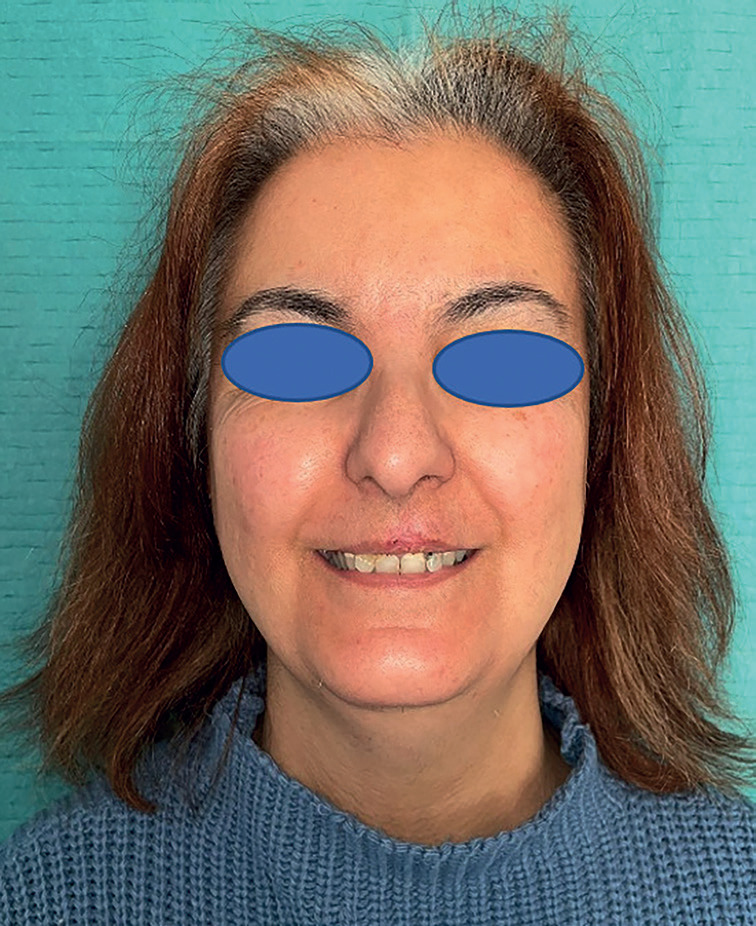
Preoperative picture of the patient.

We describe the case of a 46-year-old deaf woman with no other background of interest who suffers from a lesion on the upper lip of 3 months of evolution and progressive growth. No past history of tobacco or alcohol. She presented a 15mm hard consistency, poorly defined borders, erythematous lesion with central ulceration at the level of the philtrum and extension to nasal base (Fig. 1).

The CT scan provided no information on local extension due to the presence of dental amalgams, with no evidence of cervical ganglionar disease. An incisal biopsy was done, with the result of microcystic adnexal carcinoma that affected the underlying muscular tissue.

She underwent on a resection surgery with 1cm margins, performing a subtotal resection of the upper lip. Reconstruction was performed with a local advancement flap. A nasogastric tube was placed to avoid infection and reduce lip mobility; which was taken off 7 days later. The patient had an adequate evolution and was discharged from hospital the following day. Prophylactic antibiotic therapy with amoxicillin-clavulanic acid was prescribed for 7 days.

**Figure 2:**
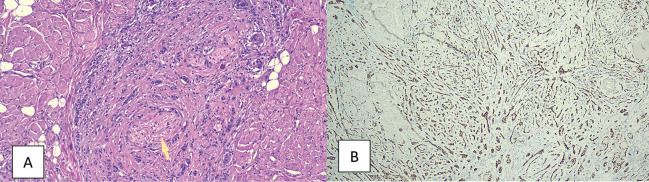
A. HE (10X). Perineural and striated muscular infiltration by the epithelial neoplasm which grows concentrically forming thin cords in several neural tracts (arrowe). Cellular details can be seen, small and medium-sized cells with small eosinophilic cytoplasm and enlarged oval nuclei with dense chromatin. B. IHC (5X) for p63. Nuclear positivity for p63 showing the infiltrative architectural pattern of tumour cells between muscle and adipose tissue.

In the definitive anatomopathological study, the following was observed: Adnexal epithelial neoplasm with imprecise borders, poorly delimited, non-encapsulated, growing in plaque-like formation from the superficial dermis into the adipose tissue. Frequent images of perineural invasion consisting of well-defined solid nests and cords made up of cells with large, poorly defined cytoplasmic cytoplasms, poorly demarcated broad eosinophilic cytoplasm and oval nuclei of fine chromatin generally isomorphic without nucleolus with abrupt trichilemmal keratinisation. The epidermis shows no tumour infiltration or alterations in any of its layers. The surgical resection margins are respected, (Fig. 2).

The case was presented in the tumour committee of our centre with de decision of no adjuvant therapy and strict follow-up.

**Figure 3:**
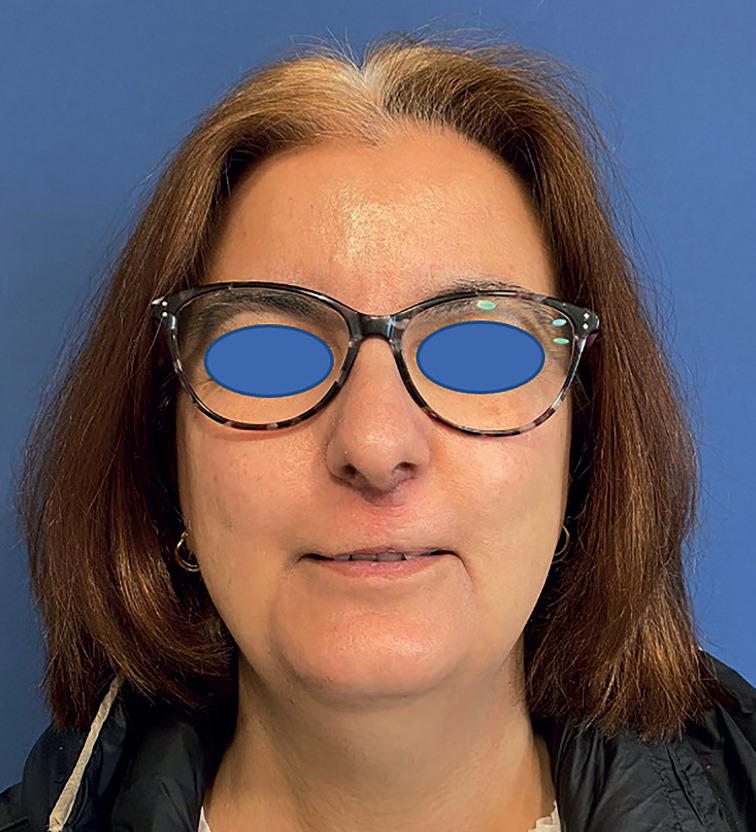
Postoperative picture.

There were no postoperative complications. The patient presented adequate aesthetic and functional results. No signs of recurrence 2 months after surgery. She will continue with biannual follow-up, (Fig. 3).

## Discussion

Microcystic adnexal carcinoma is an extremely rare malignant tumour that often affect middle-aged caucasic, female patients (2,5). Its usual presentation form is a solitary papule or plaque affecting the central face, although other localisations have been described (5). We describe the case of a 46-year-old caucasic woman who presented a solitary erythematous papule affecting the philtrum and upper lip.

UV radiation, ionizing radiation and immunosuppression have been described as possible risk factors, without solid evidence found as is seen in Worley *et al*. systematic review (5). Our patient had no history of previous immunosuppression or ionizing radiation.

This entity has local aggressive nature, characterized by small nests and strands of cells in deep dermis and perineural-invasion images, absent in superficial tissue (10). That is the reason there is a high rate of underdiagnosed cases with superficial shave biopsy, and the need of an incisional biopsy (1,5). A CT or MRI should be done to study de extension of locoregional illness, but there is no recommendation of extension image studies due to its very low metastatic rate (5,9).

The first-line treatment is surgery, without consensus in the preferred technique or security margin. Mohs micrographic surgery has been described as the technique of choice, due to the microscopic negative margin confirmation during the resection(11). Nevertheless, some studies have described significant recurrence rates with this technique (6). Other surgical opcion is the complete resection with free surgical margins. In the systematic review of Worley *et al*. (5) they recommend a 2cm security margin if circumferential resection is done. However, other studies as the one of Kühn *et al*. affirm that 1cm margin is enough (8); but others are even more aggressive, with 3cm margin recommendation (7). In our patient we decided a resection of the lesion with 1cm circumferential margin and immediate reconstruction with local advancement flap.

The spread to lymphatic cervical nodes and metastasis is very uncommon, with no recommendation of lymph node biopsy if the image studies are negative, no recommendation of lymph node dissection nor extension image studies (5,7). The recurrence is very common even with correct surgical treatment, with 10-year recurrence rate between 12-18% (7,10). There is no strong evidence to recommend adjuvant radiotherapy, although it may be considered in recurrent tumours, near or affected margins after resection (2,5,9). The role of primary chemo or/and radiotherapy remains unclear; Pugh *et al*. (7) described a case of microcystic adnexal carcinoma of the upper lip treated with radiotherapy and reirradiation after a 48-month recurrence without any signs of clinical recurrence after. Kim *et al*. (4) described a case of microcystic adnexal carcinoma treated with chemoradiotherapy without recurrence in 6 years follow-up.

Microcystic adnexal carcinoma usually appears as a papule or plaque in central face. An incisional biopsy is needed to avoid misdiagnosis, as the epidermis remains unaffected. Surgery is the first-line treatment, with high recurrence rates. Due to its rarity there is no consensus on the best management and follow-up. It is an entity that should be taken into consideration in the differential diagnosis, but it is necessary studies with larger number of patients in order to make consensus guidelines for its diagnosis and treatment.
